# Modeling Metabolic Interactions in a Consortium of the Infant Gut Microbiome

**DOI:** 10.3389/fmicb.2017.02507

**Published:** 2017-12-14

**Authors:** Francisco Pinto, Daniel A. Medina, José R. Pérez-Correa, Daniel Garrido

**Affiliations:** Department of Chemical and Bioprocess Engineering, School of Engineering, Pontificia Universidad Católica de Chile, Santiago, Chile

**Keywords:** metabolic interaction, gut microbiome diet, prebiotics, mathematical modeling, fructooligosaccharides (FOS)

## Abstract

The gut microbiome is a complex microbial community that has a significant influence on the host. Microbial interactions in the gut are mediated by dietary substrates, especially complex polysaccharides. In this environment, breakdown products from larger carbohydrates and short chain fatty acids are commonly shared among gut microbes. Understanding the forces that guide microbiome development and composition is important to determine its role in health and in the intervention of the gut microbiome as a therapeutic tool. Recently, modeling approaches such as genome-scale models and time-series analyses have been useful to predict microbial interactions. In this study, a bottom-up approach was followed to develop a mathematical model based on microbial growth equations that incorporate metabolic sharing and inhibition. The model was developed using experimental *in vitro* data from a system comprising four microorganisms of the infant gut microbiome (*Bifidobacterium longum* subsp. *infantis*, *Lactobacillus acidophilus, Escherichia coli*, and *Bacteroides vulgatus*), one substrate (fructooligosaccharides, FOS), and evaluating two metabolic products (acetate and lactate). After parameter optimization, the model accurately predicted bacterial abundance in co-cultures from mono-culture data. In addition, a good correlation was observed between the experimental data with predicted FOS consumption and acid production. *B. infantis* and *L. acidophilus* were dominant under these conditions. Further model validation included cultures with the four-species in a bioreactor using FOS. The model was able to predict the predominance of the two aforementioned species, as well as depletion of acetate and lactate. Finally, the model was tested for parameter identifiability and sensitivity. These results suggest that variations in microbial abundance and activities in the infant gut were mainly explained by metabolic interactions, and could be properly modeled using Monod kinetics with metabolic interactions. The model could be scaled to include data from larger consortia, or be applied to microbial communities where sharing metabolic resources is important in shaping bacterial abundance. Moreover, the model could be useful in designing microbial consortia with desired properties such as higher acid production.

## Introduction

The human colonic microbiome is a complex microbial community that has a significant impact on host health. This is a diverse community that reaches high cell densities and includes four dominant phyla (*Bacteroidetes*, *Firmicutes*, *Actinobacteria* and *Proteobacteria*) (Lozupone et al., 2012; Qin et al., 2013). The gut microbiome coexists with the host and deploys important functions that impact host metabolism and gut physiology (Rajilić-Stojanović and de Vos, 2014). Even though its composition is variable among people (Goodrich et al., 2014), the functions these microorganisms performed are basically conserved (Lozupone et al., 2012; Qin et al., 2013). In certain cases, imbalances in the composition of the microbiome are a contributing factor to the onset of inflammatory bowel diseases as well as autoimmune and metabolic (Greenblum et al., 2012; Sevelsted et al., 2015; Marchesi et al., 2016; Tamburini et al., 2016; Cox et al., 2017). How the microbiome assembles in the first months of life appears to be important later in life (Tamburini et al., 2016). One important factor shaping the early microbiome is the type of feeding (Qin et al., 2013; Rajilić-Stojanović and de Vos, 2014). Human breast milk contains large amounts of oligosaccharides (HMO), which are selectively utilized by beneficial gut microbes. *Bifidobacterium* species such as *B. longum* subsp. *infantis* display multiple adaptations to utilize these substrates (Thomson et al., 2017). Lactobacilli are also abundant in the infant gut microbiome (Bäckhed et al., 2015). In contrast, formula-fed infants have a distinct microbiome composition, not dominated by *Bifidobacterium* and with a higher representation of members of *Bacteroides* (*B. fragilis*, *B. vulgatus*) and *Enterobacteria* (*Escherichia coli*, *Klebsiella* spp.) (Bäckhed et al., 2015). The activity of these microbes results in high amounts of acetate and lactate in infant feces, resulting in an acidic pH (Cinquin et al., 2004; Tamburini et al., 2016).

Microbial interactions are important for the assembly and functioning of the gut microbiome. Dominant ecological interactions found in the gut microbiome are competition and cooperation (Faust and Raes, 2012). These interactions broadly represent the sum of all physical, chemical and microbiological activities that microorganisms exert upon others (Roume et al., 2015; Vogt et al., 2015; Hecht et al., 2016; Rakoff-Nahoum et al., 2016). Considering that diet is a major driver guiding gut microbiome composition, microbial interactions are influenced by dietary compounds (Cameron et al., 2014; Medina et al., 2017; Tuncil et al., 2017). Cross-feeding of fermentation breakdown products of the microbiome appears to be common among gut species (Rogowski et al., 2015). This has been shown for example in the utilization of mucin and sialylated milk oligosaccharides between *B. bifidum* and *B. breve* (Egan et al., 2014a,b), or during fructan consumption between bifidobacteria and butyrate- producing bacteria (Moens et al., 2016). Cross-feeding is also observed when metabolic end products from one microorganism, such as amino acids or short chain fatty acids (SCFA), are used by another microorganism (Egan et al., 2014a; Moens et al., 2016). For example, lactate and acetate are end products of lactic acid bacteria, which could be utilized by butyrate-producing bacteria such as *Faecalibacterium prausnitzii* and *Eubacterium rectale* (Louis and Flint, 2017).

Modeling-based approaches have been recently developed to study and predict the composition and interactions in the gut microbiome (Magnúsdóttir et al., 2016). These include ecological-statistic models, genome-scale metabolic reconstructions (GSM) and ordinary differential equation (ODE)-based kinetic models (Trosvik et al., 2010a; Kettle et al., 2015). A Generalized Additive Model (GAM) (Hastie and Tibshirani, 1990) consists of a statistic regression technique that has been used in time-series analysis of ecological data to characterize and estimate cross-feeding and competition between microorganisms. GAMs do not need any assumption about functional relationships in the group for its formulation. However, they could be affected by overfitting when many parameters are needed for matching the data (Wood, 2008; Trosvik et al., 2010b). GAMs usually require a post cross-validation process to curate the model (Ward, 2014). After proper calibration and validation, these models provide accurate predictions by interpolation (Trosvik et al., 2010b).

Lately, GSMs have been successfully applied to explore microbial interactions among gut microbes (Magnúsdóttir et al., 2016). They require an extensive database for reconstruction, editing and gap-filling of full metabolic pathways (Thiele et al., 2014). Several techniques based on orthology, topology and stoichiometry of biological reactions facilitate the draft design and curation process (Thiele and Palsson, 2010). Characteristic features of the species to be reconstructed must be first identified (Kanehisa, 2006). After curation and defining specific environments and constraints, microbial interactions can be obtained for a few species (Thiele et al., 2013).

Recently, a kinetic model constructed from experimental data of gut microbes in a bioreactor was presented, aimed to model the dynamic behavior of the gut microbiome (Kettle et al., 2015). The analysis required a metabolic pathway input and a matrix describing the compounds produced during the fermentation, to generate an ODE system for simulation of microbiome abundance (Walker et al., 2011). Here, microbiome complexity was simplified assigning gut microbes to ten bacterial functional groups (BFGs), based on metabolic properties such as similar breakdown of complex substrates or similar SCFA production or consumption patterns (Kettle et al., 2015). The model showed a good fit with experimental data, which corresponded to a continuous flow bioreactor inoculated with human fecal microbiota.

In order to help understanding the forces dominating gut microbiome structure and composition, here we developed and assessed a mathematical model based on microbial growth equations, taking into account metabolic interactions among bacteria. We focused on the interactions of four gut microbes, *Bifidobacterium longum* subsp. *infantis*, *Lactobacillus acidophilus*, *Bacteroides vulgatus* and *Escherichia coli*, during their growth *in vitro* using fructooligosaccharides (FOS) as substrate. FOS is a well studied prebiotic with degree of polymerization of fructose of 3–6 units (Roberfroid et al., 2010). Experimental data was obtained from co-culture experiments, which were used later to construct and calibrate the model, including the impact of metabolic inhibition or stimulation on bacterial growth. The model was finally validated using additional experimental data of the consortium of the four species on FOS using a biological reactor.

## Materials and Methods

### Microorganisms and Media

Microorganisms used in this study were obtained from the UC Davis, Department of Viticulture and Enology Culture Collection (*L. acidophilus* ATCC 4356, *B. infantis* ATCC 15697, *Escherichia coli* K12), and the American Type Culture Collection (*Bacteroides vulgatus* ATCC 8482; Manassas, VA, United States). Bacteria were, respectively, cultured at 37°C for 24 h in de Man–Rogosa–Sharp (MRS), MRS supplemented with 0.05% L-cysteine-HCl (Loba Chemie, India), LB broth, or Reinforced Clostridium Medium (Becton-Dickinson) supplemented with 1 g/L L-cysteine. All bacteria excepting *E. coli* were routinely grown under anaerobic conditions in an anaerobic jar (Anaerocult, Merck, Germany) with anaerobic packs (Gaspak EM, Becton Dickinson). All media were pre-reduced in an anaerobic jar overnight before inoculation, and prior to each assay bacteria were sub-cultured twice.

### Co-culture Batch Experiments

Combinations of *L. acidophilus* (La), *E. coli* (Ec), *B. vulgatus* (Bv) and *B. infantis* (Bi) were prepared in co-culture experiments. Culture media used was a modified version of previously described ZMB (Zhang et al., 2009), which was supplemented with hemin (0.01 g/L, Sigma–Aldrich, St. Louis, MO, United States) and L-cysteine-HCl (0.5 g/L, Sigma–Aldrich, St. Louis, MO, United States). Single amino acid groups in ZMB were replaced by Bacto-Tryptone (at 28 g/L). Carbon sources used were either lactose (10 g/L; Lyngby, Denmark) or FOS (10 g/L; Raftilose Synergy 1, Orafti, Malvern, PA, United States) as carbon source. Single cultures of *B. infantis* (Bi), *B. vulgatus* (Bv), *E. coli* (Ec) and *L. acidophilus* (La); and co-cultures BiBv, BiEc, BiLa, BvEc, BvLa and EcLa were prepared. An experiment with all bacteria (All) and a negative control with no bacteria were included. Fresh overnight cultures of each microorganism were washed in sterile mZMB, and 1 mL of each overnight culture was used to inoculate 10 mL of mZMB containing FOS. This experiment was performed in duplicate. Volumes of 200 μL of inoculated mZMB were placed in 96 well sterile microplates, covered with 30 μL of sterile mineral oil, and incubated in anaerobic jars at 37°C for either 24, 48, or 72 h. In parallel, growth was monitored every 12 h in a microplate reader (Tecan Infinite M200 PRO, Switzerland). Samples were recovered from each microplate and centrifuged at 12000 ×*g* for 2 min. Pellets and supernatants were stored at -20°C until use.

### Quantification of Bacterial Abundance by qPCR

Total DNA from each sample was purified using the UltraClean^®^ Microbial DNA Isolation Kit (Mo Bio Laboratories, Carlsbad, CA, United States), following manufacturer instructions and using a Disruptor Genie (Scientific Industries, Inc., Bohemia, NY, United States). Extracted DNA was quantified using a NanoQuant Plate in the Tecan Infinite M200 PRO plate reader, and diluted to 1 ng/μL to be used in qPCR reactions. For qPCR we used 0.2 μM of the following primers: for Bv, *Bacteroidetes* primer F (5′-GGTGTCGGCTTAAGTGCCAT-3′) and *Bacteroidetes* primer R (5′-CGGACGTAAGGGCCGTGC-3′); for Bi, Blon_0883F (5′-AGTTCGGCTCCAAAGACCTG-3′) and Blon_0883R (5′-CATGCCTCGATACGGTCGAA), targeting an ABC solute binding protein; for Ec, Eco1457F (5′-CATTGACGTTACCCGCAGAAGAAG) and Eco1652R (5′-CTCTACGAGACTCAAGCTTGC-3′) (Kassinen et al., 2004); and for La, LACTO_F (5′-TGGAAACAGRTGCTAATACCG-3′) and LACTO_R (5′-GTCCATTGTGGAAGATTCCC-3′) (Bartosch et al., 2004). qPCR reactions were performed using the qPCR PowerUp SYBR Green Master Mix in MicroAmp Fast Optical plates (Applied Biosystems, United States), and using a StepOnePlus Real-Time PCR System (Applied Biosystems, United States). Reactions were carried out for 2 min at 50°C, 2 min at 95°C and 40 cycles of 3 s at 95°C and 30 s at 62°C. Absolute quantification was performed including a standard curve using DNA from a pure culture of each species, with dilutions starting from 1 ng/μL to 0.1 pg/μL. To convert bacterial DNA concentrations into cell genome numbers, the following equation was used (equation 1).

$$\text{Cell copies/mL}=\frac{\text{Avogadro N}{}^{\circ}\;(\text{1/mol})\cdot \text{DNA quantity }\left(\text{g/ml}\right)\cdot \text{Genome 16S copy number}}{\text{Genome size }(\text{pb})\cdot \text{660}\left(\frac{\text{g}}{\text{mol}}\right)}$$

### Batch Bioreactor Culturing

Four independent batch co-culture experiments were performed in a 250 mL bioreactor (Mini-bio Applikon Biotechnology, Netherlands), using mZMB as culture media supplemented with FOS at 1%. In these experiments, the four microorganisms (Bi-La-Ec-Bv) were inoculated at an initial OD_630_ of 0.05. The bioreactor has two six-bladed Rushton turbines and operated at 100 rpm. The temperature was set at 37°C and the pH was maintained at 5.5 with automatic injection of 3N HCl and 3N NaOH. The dissolved oxygen concentration was set at 1 ppm by purging N_2_ (99.99% grade) before inoculation and during the lag phase. The foam level was controlled adding 100 μL antifoam in the inoculum (Polydimethylsiloxane base, Winkler, Chile). Two milliliter from the bioreactor were obtained every 2 h and centrifuged at 4000 ×*g* for 5 min. Supernatants were stored at -20°C for carbohydrate and SCFA quantification. Pellets were stored for DNA extraction, quantified and diluted to 10 ng/μL for qPCR assays as described above in an AriaMx Realtime PCR System (Agilent Technologies, Santa Clara, CA, United States).

### Sample Analysis

Total carbohydrate quantification was performed using the phenol-sulfuric acid method (Tuomivaara et al., 2015). Acetate and lactate were quantified by HPLC using an Aminex HPX-87H ion exchange carbohydrate-organic acid column (Bio-Rad, United States) at 35°C with a flow rate of 0.450 mL/min (H_2_SO_4_ 5 mM, mobile phase) on a LaChrom L-700 HPLC system (Hitachi, Japan), equipped with a Diode Array and a Refractive Index detectors as described previously (Mendoza et al., 2017).

### Model Development

The equations used in the model are described in the Model development section in Supplementary Material. The model, the parameter identifiability and sensitivity analysis codes are also presented in Supplementary Material. As input for the determination of the parameters, mono-culture and paired co-culture abundance data are required, in addition to an estimation of acetate and lactate produced and carbohydrate consumed under these conditions. To simplify the analysis, some assumptions were taken into account: (a) an inhibition term was added to Monod kinetics (Model development, Supplementary Material); (b) a microorganism will prefer the consumption of the main carbon sources (glucose, lactose), over other intermediates produced during the fermentation; (c) the ability of a microorganism to produce or consume an intermediate was determined from its metabolic pathway and the literature, and later confirmed experimentally in mono-cultures.

## Results

### Model Description

**FIGURE 1:**
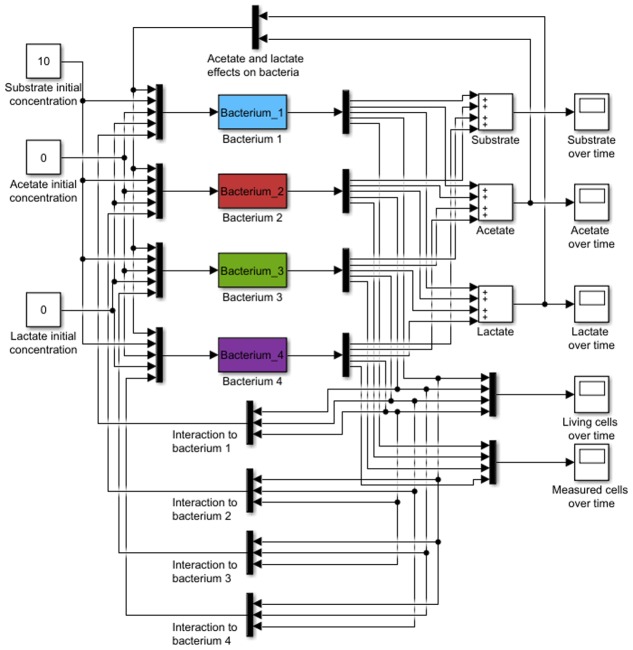
Model general representation. Initial substrate and product concentrations and lag phase are used as input (black bars). Microbial growth, consumption, and acid production are considered to interact with other bacteria. Final outputs are observed substrate, acids, and biomass.

In this work a kinetic black-box model was developed, aimed to predict the abundance of a bacterial population, substrate consumption and SCFA production, based on mono and co-culture data (**Figure 1**). The model is based on microbial growth equations, but it also considers the metabolic influence of one microorganism on another. This could be considered as a feedback control mechanism (**Figure 1**).

### Parameter Settings in Mono-culture

For single microorganisms, the general model consisted of 5 ODEs (Equations 2, 4, 5, and 6 in Supplementary Material), 17 parameters and constitutive Monod-like inhibition equations (Sacher et al., 2011). Mono-culture parameters (**Table 1**) were set as described in the Parameter fitting section in the Supplementary Material. 96 well-plates mono-cultures of Bi, Bv, Ec and La were prepared, in a semi-synthetic media (mZMB) and using FOS as the sole carbon source. Bacterial abundance, FOS consumption and acetate and lactate produced were measured to fit model parameters. An average of eight parameters were set for each bacterium (**Table 1**), which were found by the optimization task. The calculated error in the assay is shown in Supplementary Table S1. For any microorganism and under all conditions, parameter *K*_s_ (half-velocity constant) appeared insensitive.

**Table 1:** Parameters found via scatter search in mono-culture and then used in co-culture optimization.

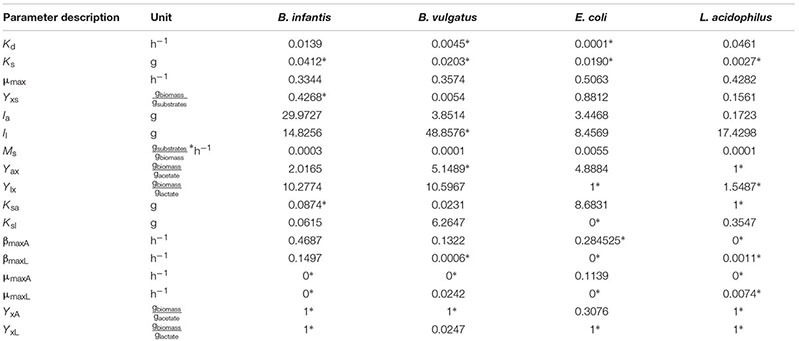

*Set parameters are indicated by (^∗^).*

### Paired Co-culture and Parameter Fitting

**FIGURE 2:**
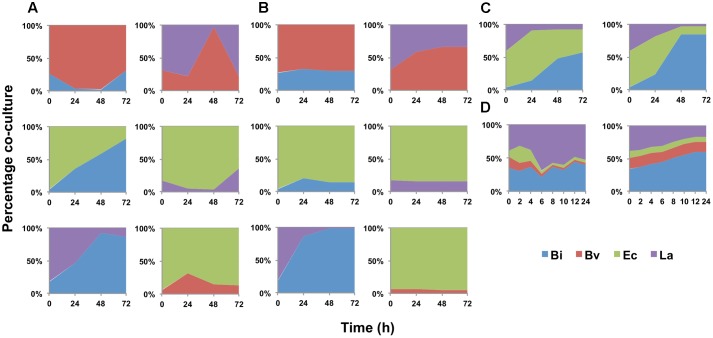
Changes in bacterial population during growth on FOS, expressed as percentage of the co-culture in time. **(A)** experimental data of co-cultures; **(B)** model estimation of abundance in co-cultures; **(C)** abundance of the four-species co-culture in microplates, experimental (Left) and estimated by the model (Right); **(D)** abundance in the four-species co-culture in the bioreactor during growth on FOS, experimental (Left) and estimated by the model (Right).

The model was later expanded to include the metabolic interaction between two microorganisms. This model consists of 7 ODEs, 17 parameters per bacteria and two interaction parameters per co-culture. Every parameter not calibrated in mono-culture was set in this step. In order to fit the co-culture parameters, all paired combinations of microorganisms were cultured in FOS and analyzed as described above. **Figure 2A** shows the percentage of change in abundance for all six paired combinations, determined experimentally. As a comparison, **Figure 2B** shows these percentage changes according to the fitted models. Most of the times, the model was able to predict well the changes in abundance in all co-cultures. Experimentally, initial Ec cell numbers were higher than the other microorganisms. However, during growth Bi and La recovered in part their levels compared to Ec (**Figures 2A,B**). Co-culture data allowed the prediction of Bv predominance over La and Bi during growth on FOS, which was also observed experimentally.

**FIGURE 3:**
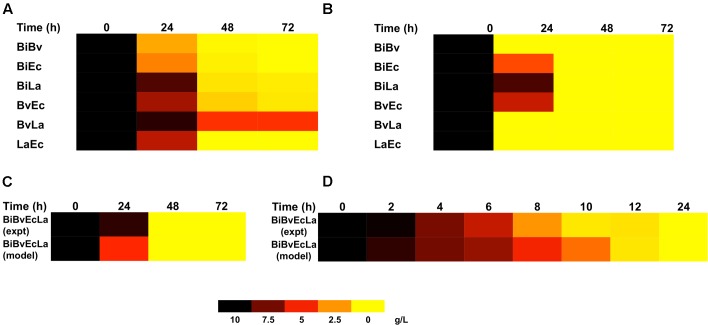
Heat map representing FOS concentration in co-cultures and its prediction by the model. **(A)** FOS concentration in paired co-cultures. **(B)** Model estimation of FOS concentration in paired co-cultures; **(C)** experimental and predicted FOS concentration in the four-species co-culture in microplates; **(D)** experimental and predicted FOS concentration in the four-species co-culture in the bioreactor.

**Figures 3A,B** compares the experimental consumption of FOS by the co-cultures with the values simulated with the fitted model. Most experimental and simulated combinations showed total carbohydrate depletion between 24 and 48 h. In general the model indicated a faster consumption compared to experimental data. One important exception was the BvLa paired co-culture, in which not all of the carbohydrate was consumed. This behavior was not captured by the model, which assumed that since both bacteria reached 100% consumption in single culture, the same rule should apply to their combination.

**FIGURE 4:**
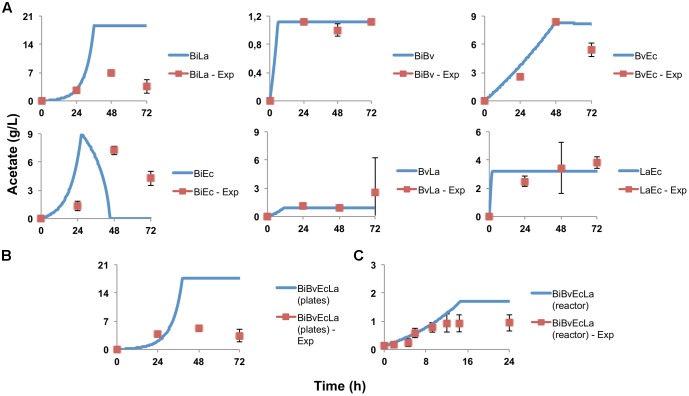
Acetate production and estimation by the model. **(A)** Experimental data in paired co-cultures, compared to values predicted by the model; **(B)** experimental and predicted acetate values of the consortium in microplate assay; **(C)** experimental and predicted acetate values of the consortium in the bioreactor.

**Figure 4A** shows the concentration of acetate produced over time. In certain cases the model predicted the experimental behavior of acetate production. Bi combinations displayed larger acetate amounts compared to other co-cultures, and in certain cases the model predicted higher values than what was observed. Interestingly, the model predicted that acetate production in co-culture BiEc will have a peak and later decrease. This was also observed experimentally, but at a different time and different intensity (**Figure 4A**). These results indicate that Bi growth is an important parameter for sensitivity assays.

**FIGURE 5:**
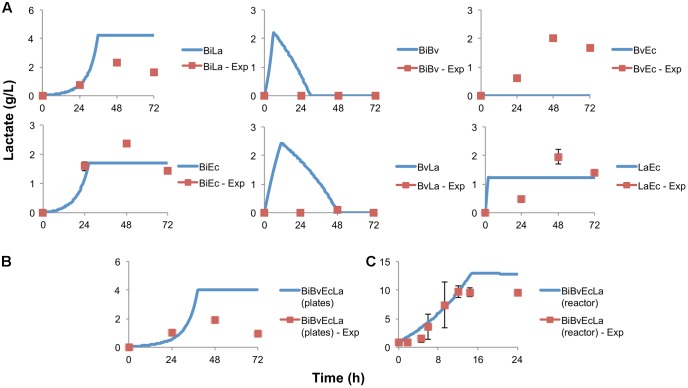
Lactate production and estimation by the model. **(A)** Experimental data in paired co-cultures, compared to values predicted by the model; **(B)** experimental and predicted lactate values of the consortium in microplate assay; **(C)** experimental and predicted lactate values of the consortium in the bioreactor.

**Figure 5A** displays the concentration of lactate in co-cultures. A good agreement between observed and predicted data was obtained in co-cultures BiLa, BiEc and LaEc. Combinations BiBv and BvLa were predicted to produce lactate because of Bi and La activities; however, lactate amounts were negligible and not reproduced well by the model. In addition, BvEc co-culture showed production of lactate, but the model assumptions and structure did not consider this situation. The error calculated (equation 10 in Supplementary Material) for the parameter fitting process is shown in Supplementary Table S1.

The parameters determined in paired co-cultures are shown in **Table 1**. The interaction parameters in **Table 2** indicate the influence of one microorganism on another’s growth rate. A negative value indicates that one microorganism favors another’s growth, while a positive term indicates inhibition. Values near 0 suggest a greater interaction effect, while values near the limit indicate there is no effect on the other bacteria. A strong inhibition was found from Ec to Bv, and in general the effects observed were positive or neutral.

**Table 2:** Interaction parameters (ef_ji_ in equation 8, Supplementary Material) found in co-cultures by the model.

	*B. infantis*	*B. vulgatus*	*E. coli*	*L. acidophilus*
*B. infantis*	–	99.99	16.33	45.05
*B. vulgatus*	-40.52	–	0.16	1.99
*E. coli*	-37.56	-57.14	–	-26.61
*L. acidophilus*	-70.23	-32.74	-99.99	–

*Negative values indicate growth stimulation, and positive indicates a negative effect on growth. Values near 0 indicate a stronger effect.*

### Model Validation Using Bacterial Consortia

Finally, the model was validated using independent experimental data from co-culture of the four microorganisms using FOS as the sole carbon source. The experiment was set in microplates and analyzed as discussed above. To test the validity of the model in another set-up, the consortium was additionally cultured on FOS in a 250 mL pH/oxygen controlled stirred bioreactor. This batch system offers a much more controlled and reproducible anaerobic environment, which also provides much faster growth compared to microplates.

**Figure 2C** shows percentage abundance data obtained for each member of the consortium in microplate assays. The initial levels of Bv were much lower compared to the other three microorganisms. Interestingly, the amounts of La, Ec and Bi in the well-plates cultures were closely predicted by the model. Under these conditions, Bi dominated the co-culture using FOS, followed by La. A good prediction was also observed for the total carbohydrate concentration in spent media (**Figure 3C**). Finally, the amounts of acetate and lactate appeared overestimated by the model (**Figures 4B**, **5B**).

As expected, growth of the consortium in the bioreactor resolved in a shorter time compared to the assays above (**Figure 2D**). Therefore, time was linearly adjusted for comparison and integration in the model. As in microplates, we observed a predominance of Bi and La. This observation was sustained during the course of the fermentation. Interestingly, the model was also able to predict this predominance (**Figure 2D**). In addition, both the model and data showed a full consumption of FOS at 12 h (**Figure 3D**). Finally, a good agreement of acetate and lactate amounts between the experimental evidence and the model was obtained (**Figures 4C**, **5C**). Since La was a good competitor during growth on FOS in the bioreactor, lactate concentrations appeared higher compared to previous experiments (**Figures 5A–C**). The parameters that define the production of lactate and acetate in Bi appear to be important in the four-bacterium co-culture, considering the predominance of Bi.

**FIGURE 6:**
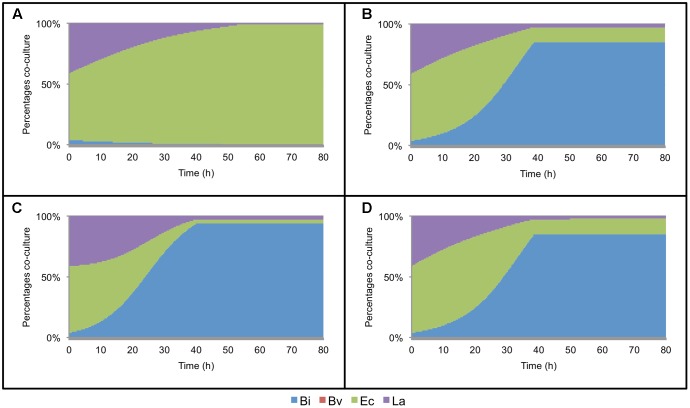
Simulation of the effect of a bacteriostatic agents on the consortium. These agents are simulated to be directed and inhibit the growth of each member of the consortium. **(A)** *Bifidobacterium infantis* is unable to grow; **(B)** *Bacteroides vulgatus* is unable to grow; **(C)** *Escherichia coli* is unable to grow; **(D)** *Lactobacillus acidophilus* is unable to grow.

Finally, we performed a simple additional simulation to test the prediction capabilities of the model where a bacteriostatic agent is used against each member of the consortium (**Figure 6**). In every co-culture where Bi was able to grow, it predominated over the others (**Figures 6B–D**). On the other hand, if Bi was inhibited, Ec predominated in the co-culture (**Figure 6A**).

### Parameter Identifiability Analysis

**FIGURE 7:**
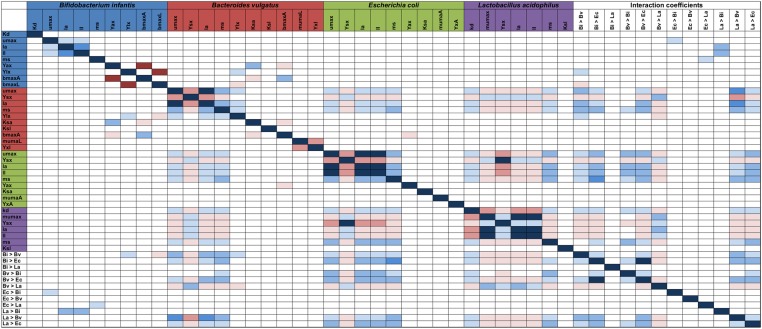
Parameter model identifiability. Correlation values between each parameter in the model was calculated (for each microorganism including interaction). Only >|0.95| values are highlighted; red values are inversely correlated, while blue values are directly correlated. Parameters on both axes are indicated in **Table 1**.

Parameter identifiability was used to find correlations between parameters (Parameter identifiability in Supplementary Material). This analysis is important for further reducing the number of fitted parameters by setting one of them and defining the other as a function. Inspection of the parameter covariance matrix is one way to find which parameters allow the model to be identifiable. As shown in **Figure 7**, highlighted cells display a high correlation (positive or negative). Usually parameters inside a cluster have a high correlation. In this case, this could be observed for all parameters from the same microorganism. For example, production of acetate and lactate in Bi are directly correlated, while some correlations between microorganisms were found. La’s parameters (*Y*_sx_ – biomass yield, μ_max_ – Maximum growth rate, *I*_a_ – Acetate inhibition constant, *I*_l_ – Lactate inhibition constant) are inversely correlated to Ec bacterial parameters such as growth and inhibition constants. This suggests that the higher the La growth, the lower the *E. coli* biomass yield and higher inhibition. Several parameters associated to Bv growth were mostly directly correlated to Ec growth, indicating a more neutral or cooperative interaction.

### Parameter Sensitivity Analysis

**FIGURE 8:**
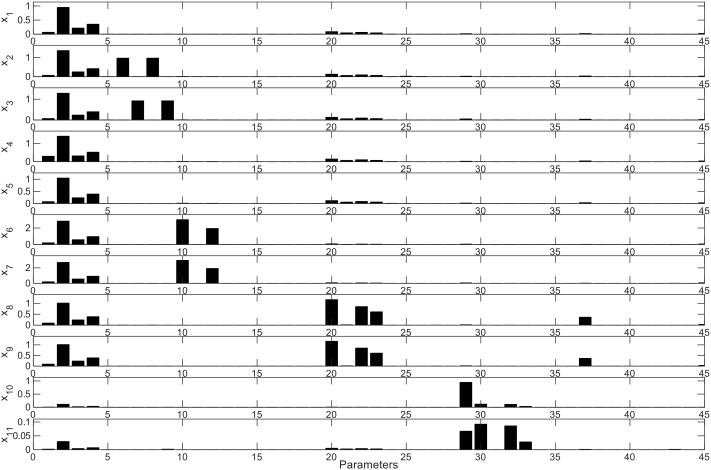
Model parameter average sensitivity. Sensitivity (*Y*-axis) of each parameter (*X*-axis) for every ODE is shown. *x*_i_ represents every ODE described in the model (*x*_1_ = dS/dt, *x*_2_ = dA/dt, *x*_3_ = dL/dt, *x*_4_ = dX_1_/dt, *x*_5_ = dX_1m_/dt, *x*_6_ = dX_2_/dt, *x*_7_ = dX_2m_/dt, *x*_8_ = dX_3_/dt, *x*_9_ = dX_3m_/dt, *x*_10_ = dX_4_/dt, *x*_11_ = dX_4m_/dt), where S: substrate; A: acetate; L: lactate. X: live biomass; *X*_m_: total biomass. Parameters are in the same order in **Figure 6**.

**FIGURE 9:**
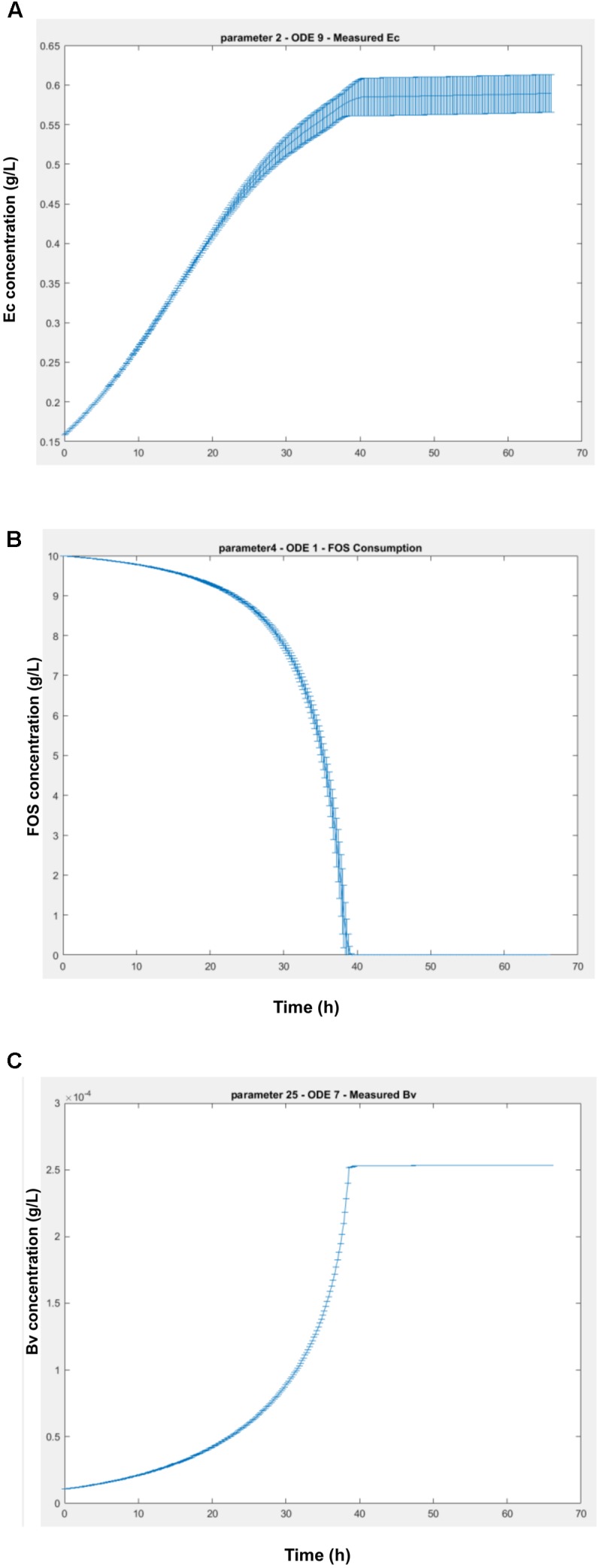
Variation of the ODEs values (g/L) over time due a 5% change in the parameters in 5000 iterations. **(A)** worst case scenario, with parameter 2 (Bi’s μ_max_) affecting measured Ec ODE; **(B)** a change in kinetics scenario, with parameter 4 (Bi’s lactate inhibition constant), affecting FOS consumption; **(C)** a non-sensitive parameter (measured Bv ODE), for example to Ec substrate yield.

This analysis allows the determination of the influence of every parameter in each differential equation of the model. As shown in **Figure 8**, the effects of the parameters initially set are important in every ODE, due to the fact that Bi appears as the dominant microorganism in the consortium (**Figures 2C,D**). Specifically, the second parameter of the model (Bi’s μ_max_) has the highest influence on every other microorganism and their metabolic equations. Parameters *K*_3_ and *K*_4_ (Bi’s inhibition constants of acetate and lactate) also display a large influence on other microorganisms. In order to analyze the effects of the sensitive parameters found in the previous assay, **Figure 9** shows the average and standard deviation after 5000 iterations of randomly changing a parameter by 5% in its amount. The strongest effect of changing the value of Bi’s μ_max_ is on Ec cell numbers (**Figure 9A**), variable that can vary around 4% the value. On the other hand, a change in a parameter could also imply an advance or delay in the kinetics. **Figure 9B** shows the change in the FOS consumption kinetics due to effects of higher or lower values of Bi’s lactate inhibition constant. Here we observed that changing the parameter only altered the dynamics of the ODE. Finally, **Figure 9C** shows the last case found in the sensitivity analysis, a parameter that is not sensitive to any differential equation. For example, measured Bv was not affected even after changing 50% parameter 25 (Ec substrate yield *Y*_sx_).

## Discussion

The gut microbiome is a complex microbial community that modulates several host responses. This connection to host health makes it important to understand what forces guide microbiome composition and cause it to drift to an altered or dysbiotic microbiome (Cox et al., 2017). The interest in determining and predicting key factors in the establishment and maintenance of the gut microbiome is the major goal of several works (Trosvik et al., 2010a; Greenblum et al., 2012; Kettle et al., 2015; Shashkova et al., 2016).

Diet is a major modulator of the composition of the gut microbiome, and the nature of these substrates probably dictates which species predominate. In this study we evaluated if a mathematical model capturing metabolic interactions is able to recapitulate the composition and functions of a consortium of species of the gut microbiome. For this, we chose four representative bacteria of the infant gut microbiome, and using experimental data from mono and co-culture, a model was developed, calibrated and validated. Using a bioreactor, the developed model was assessed in a more controlled environment.

The system was studied during growth on FOS, a major prebiotic present in infant formula (Roberfroid et al., 2010). All members of the consortium display the ability to use this substrate (Roberfroid et al., 2010), including *E. coli* which could use small amounts of mono or disaccharides found in FOS. Moreover, different molecular mechanisms for FOS consumption have been described (Barrangou et al., 2003). In general the predictions by the model followed the *in vitro* behavior of the consortium, either in paired co-cultures, and growing the four-species consortium either in microplates or in a more controlled environment such as a biological reactor. This indicates that the model is able to predict changes in the bacterial abundance using only co-culture data for calibration.

It is very possible that interactions and parameters determined in this study are dependent on which prebiotic is used. FOS are commonly added to infant formula, but in combination with galactooligosaccharides (GOS), another important prebiotic (Garrido et al., 2013). Breast milk contains large concentrations of HMO, which are also a large catalog of oligosaccharides derived from lactose (Thomson et al., 2017). Moreover, the gut epithelium is covered with a mucin layer, containing oligosaccharides that could be used as carbon source by infant gut bacteria (Tailford et al., 2015). In a more realistic situation probably all these carbohydrates contribute to shape microbial interactions in different ways, since their chemical structure selects for specific microbial strains endowed with the cognate molecular machinery for utilization. However, if metabolic interactions are key in shaping microbiome composition, we could hypothesize that a mathematical model including these interactions could predict microbiome composition when other substrates are used.

We observed a good fit between experimental data and modeling results. This suggests that inhibitions observed in certain cases could be due to acetate and lactate production, variables that were quantified and included in the mathematical model. Both the reactor and the microplates had an initial pH of 5.5, however, pH was not regulated in the latter system. Considering this, similar results in both systems could also indicate that results obtained are independent of the pH.

A general good agreement was also observed for acid production and carbohydrate consumption. For Bi in mono-cultures and co-cultures where it predominates, the amounts of acetate and lactate produced are near a 3:2 ratio (Garrido et al., 2013). This was also observed during the growth of the consortium in the bioreactor. Acetate production by Ec was overestimated by the model (0.21 g of acetate per 1 g of FOS consumed). In general Ec was thought to benefit from other microorganism activities in that it uses mono or disaccharides released to the media (**Table 2**) (Ravcheev et al., 2013; Vuoristo et al., 2015). Another possibility might be protein fermentation by Ec (Lulit and Strohl, 1990). Lactate production of La determined by the model was around 0.63 g per 1 g of substrate, a similar yield in lactose reported (Fu and Mathews, 1999).

In some co-cultures, the concentration of either acetate or lactate was overestimated. This was evident in Bi co-cultures whenever it predominated. Parameters of the model could be much better estimated in experiments with improved resolution and more frequent measurements. Since the model in co-cultures defines the intervals where the parameters are most sensitive, it is possible that an increase in the number of samples would reduce the variation of underestimated parameters. The time points where the substrate is being fully consumed are critical, and microorganisms could find another substrate for growth (for secondary fermenters) or entering to a stationary phase. Also, for *Bacteroides* and *Escherichia* cultures, the microbial concentration could be overestimated by some intrinsic pathways of these genera (Neis et al., 2015; Vuoristo et al., 2015).

Moreover, while acetate and lactate are major metabolic products in this system, a more complete picture could be obtained if the model included other metabolites. Adding more equations of utilization and inhibition by metabolites such as ethanol, propionate, butyrate and amino acids could be important. Amino acid cross-feeding between *Bacteroides* and *Lactobacillus* supports bacterial growth *in vitro* and *in silico* (Magnúsdóttir et al., 2016).

The analysis of bacteriostatic agent effects on the culture suggested that Bi should be predominant if other bacteria are inhibited. However, when Bi is inhibited, La or Bv should grow more than Ec, because of their glycolytic properties (Ravcheev et al., 2013). This is a limitation of the model, probably due to missing functions that describe the breakdown of complex carbohydrates by Bv, or the protein fermentation as a carbon source of bacteria. In addition, further work could corroborate these hypotheses by adding the respective antibiotic and measuring the same variables used in this work.

A possible application of this initial ODE-based model is that it could be used to predict microbial composition in the gut based on diet, at least in simpler microbiome communities. This work indicates that it is possible to have a good approach to this goal if metabolic interactions are included. Moreover, bacterial composition of a microbiome could eventually be optimized, for example to increase production of acetate and lactate. These two acids are important modulators of health outcomes in the gut. For example acetate has been shown to prevent pathogen colonization (Fukuda et al., 2011), and lactate in the adult gut microbiome is used by butyrate-producing bacteria (Moens et al., 2016), a health-promoting SCFA (Louis and Flint, 2017).

Finally, this model could be useful to study interactions using a more complex set of species of gut microbiome species. In general these results could be important to predict the composition of microbial communities where metabolic interactions are relevant. Considering the flexibility of incorporating product equations and growth inhibitions to the model, this model could be used to find microbial consortia with desired metabolic properties such as maximized acid production.

## Author Contributions

FP and DM performed experiments. FP developed the model. DG provided materials and reagents. JP-C and DG conceived the experiments, wrote, and edited the manuscript.

## Conflict of Interest Statement

Theauthors declare that the research was conducted in the absence of any commercial or financial relationships that could be construed as a potential conflict of interest.
