# Supplementary material for: Modeling Metabolic Interactions in a Consortium of the Infant Gut Microbiome
Source: Front Microbiol. 2017 Dec 14;8:2507. doi: 10.3389/fmicb.2017.02507 (PMC5735223; doi:10.3389/fmicb.2017.02507)
Supplement: Supplementary file 1 [file Data_Sheet_1.DOCX]

# Supplementary Material

## Model development.

The model developed describes the changes in biomass of a hypothetical standard bacterium in a batch mono-culture, as reported previously (Sacher et al., 2011). The model does not incorporate the lag phase of any bacterial growth. The model is based on the following assumptions: culture is anaerobic; temperature, pH and water activity are constant throughout the whole culture; culture is not limited in nitrogen; the carbon source is the only limiting substrate; when the main carbon source is depleted, secondary fermenters consume SCFA if this is reported in the literature; the model parameters remain constant in the model.

The measured biomass ($X_{m}$) is the total amount of dry biomass including live and dead cells (equation 2). It is estimated by the growth rate $\mu$ (equation 3).

| $\frac{dX_{m}}{dt}=\mu\cdot X$ | (2) |
| --- | --- |
| $\mu=\frac{\mu_{max}\cdot S}{K_{s}+S}\cdot\frac{1}{1+\frac{I_{A}}{K_{I_{A}}}}\cdot\frac{1}{1+\frac{I_{L}}{K_{I_{L}}}}$ | (3) |

Here, the specific growth rate ($\mu$) is modeled using Monod kinetics with product inhibition (Andrews and Noack, 1968). $\mu_{max}$ is the maximum growth rate that a bacteria will grow in the specific substrate (FOS), and $K_{s}$ is the half-velocity constant (the value of substrate when $\mu/\mu_{max} =0.5$). The other terms in the equation 3 are the concentrations of the inhibitors of the bacterial growth ($I_{A}$ for acetate and $I_{L}$ for lactate) and the respective constant of inhibition ($K_{I_{A}}$ for acetate and $K_{l_{L}}$ for lactate) (Andrews and Noack, 1968).

The live biomass ($X$) is calculated assuming a first order death rate ($k_{d}$).

| $\frac{dX}{dt}=\mu\cdot X-k_{d}\cdot X$ | (4) |
| --- | --- |

The consumed substrate (FOS) over time is defined by two principal events, the bacterial growth and the sugar uptake for maintenance (equation 5). The bacterial growth has a yield of grams of biomass generated per gram of substrate consumed ($Y_{x/s}$). The cellular maintenance is calculated assuming a first order kinetic ($m_{s}$), as described previously (Schnapp et al., 1991).

| $\frac{dS}{dt}=-\frac{\mu\cdot X}{Y_{x/s}}-m_{s}\cdot X$ | (5) |
| --- | --- |

The selected SCFA (acetate and lactate) are produced during anaerobic fermentation. These are calculated based on the production rate ($\beta$) and the yield of gram of product per produced biomass ($Y_{x/{P_{SCFA}}}$), as shown in equation 6. The equation 7 is the production rate ($\beta$) defined as a Monod kinetic without inhibition. $\beta_{max}$ is the maximum production rate of the SCFA and $K_{S}$ is the half-velocity constant.

| $\left[ \frac{dP_{SCFA}}{dt} \right]_{prod}=\frac{\beta\cdot X}{Y_{x/{P_{SCFA}}}}$ | (6) |
| --- | --- |
| $\beta=\frac{\beta_{max}*S}{K_{S}+S}$ | (7) |

For paired co-cultures, additional terms were included: microbial interactions and partial consumptions of SCFA by secondary fermenters. The interaction term was added in the growth rate equation as an inhibition product (same as SCFA), as shown in equation 8. Corresponding restrictions for any indetermination in the parameter fitting routine were added, mainly because the indetermination interval of the function is variable, and depends on the other bacteria (described in the co-culture parameter fitting section in Supplementary Material). $X_{j}\left( t \right)$is the concentration of the other bacteria, while $ef_{ji}$ is the interaction parameter of the bacteria$j$ over the bacteria $i$. Interaction ($ef_{ji}$) is considered as another inhibition product, but with a longer parameter range, to evaluate cases of cooperation and competition.

| $\mu_{i}=\frac{\mu_{max}\cdot S}{K_{s}+S}\cdot\frac{1}{1+\frac{I_{A}}{K_{I_{A}}}}\cdot\frac{1}{1+\frac{I_{L}}{K_{I_{L}}}}\cdot\frac{1}{1+\frac{X_{j}(t)}{ef_{ji}}}$ | (8) |
| --- | --- |

The partial SCFA consumption for each bacterium is added after the main substrate is depleted (equation 9). Where $\mu_{SCFA}^{i}$ is the growth rate of the bacteria $i$ with the SCFA as the carbon source, $X_{i}$ is the concentration of the living bacteria $i$, and $Y_{{X_{2}}/{P_{SCFA}}}$ is the yield of biomass using the SCFA as a carbon source. $\mu_{SCFA}^{i}$ also considers growth inhibition by SCFA and the interaction between bacteria.

| $\left[ \frac{dP_{SCFA}}{dt} \right]_{uptake}=-\frac{\mu_{SCFA}^{1}\cdot X_{1}}{Y_{{X_{1}}/{P_{SCFA}}}}-\frac{\mu_{SCFA}^{2}\cdot X_{2}}{Y_{{X_{2}}/{P_{SCFA}}}}$ | (9) |
| --- | --- |

## Parameter fitting

Model parameter fitting was performed by solving numerically the minimization of the sum of squared residual errors between the experimental and predicted data, normalized by the respective experimental data variance, $Var\left( X_{i}^{exp} \right)$, as shown in equation 10.

| $\min_{\Theta} \sum_{j}^{n} \sum_{i}^{m} \frac{\left( X_{i,j}^{exp}-X_{i,j}^{model} \right)^{2}}{Var\left( X_{i}^{exp} \right)}$ | (10) |
| --- | --- |

Where $X_{i,j}$ is the measurement $j$ of the variable $i$, m is the number of measured variables, while n is the number of measurements. $\Theta$ represents the parameter space where each model parameter combination is localized.

The optimization was solved using the SSmGO Toolbox, a scatter search routine programed for Matlab® (Egea et al., 2007; Rodriguez-Fernandez et al., 2006). The integrator used was ODE15, and relative and absolute tolerance was fixed at 1e-6. The number of evaluations was set at 20000 iterations. After this number of iterations were completed, a different initial parameter value was set, so the routine did not get stacked in a local optimum inside the plane of possible solutions.

For mono-culture, lower and upper bounds were set at 0.000001 and 100 respectively. Also, after the best result was found, it was used in the identifiability and sensitivity analysis to set the parameter. This process was iterative until the model could not fit correctly the data and the minimum of variable parameters was reached.

For paired co-cultures, four cases were analyzed, since the interaction parameter could be positive or negative. The interval $\left[ -X_{j},0 \right]$ of the interaction parameter makes the function negative, where $X_{j}$ is the concentration of the other bacteria that interact with the growing bacteria $i$. This has no biological meaning; therefore it was taken out of the parameter optimization. As in mono-culture, scatter-search was used for parameter optimization. Lower and upper bounds were set to -100 and -0.001 for the negative scenario and 0.000001 and 100 for the positive scenario respectively.

## Pre/post-regression diagnostics

As described in (Sacher et al., 2011), this diagnostic briefly consists in three analysis: Sensitivity, identifiability, and significance of the model parameters. These were programmed using Matlab®. Parameter sensitivity allows checking which parameter interferes directly in the state variables, particularly, the normalization of the calculated value is useful for comparisons (Sacher et al., 2011). Every value in the G matrix, is calculated by the partial derivative of the ODE by the parameter, and then normalized by the parameter and variable value, as shown in equation 11. This sensitivity was estimated in the time interval of 72 hours.

| $\bar{G_{ij}}\left( t \right)=\frac{\theta_{j}}{X_{i}}\frac{\partial X_{i}\left( t \right)}{\partial\theta_{j}}$ | (11) |
| --- | --- |

Parameter identifiability allows to find terms that are directly or inversely correlated locally in a given time interval. It is calculated by the correlation matrix between each parameter in the model calculated previously on the sensitivity matrix, as shown in equation 12. If two parameters are highly correlated, these parameters affect the measured variable in exactly the same way.

| $K_{ij}=correlation\left( G \right)$ | (12) |
| --- | --- |

In order to evaluate the sensitivity of the parameters in the model, a routine was made to randomly vary the value of the parameter in ±5% of the value. For the routine, 5000 iterations per parameter changed were made. After the iterations, the average and standard deviation were calculated for every ODE to identify the changes.

## Model code

The code used in monoculture is presented in the box below.

| function sys=monocult(t,x,k)  sys = zeros(5,1);  %Variables  %x(1) Substrate (FOS)  %x(2) Acetate  %x(3) Lactate  %x(4) Living bacteria  %x(5) Total bacteria    %Parameters    %k(1) kd, Death rate  %k(2) ks, Half-velocity constant  %k(3) mumax, Maximum growth rate  %k(4) Yxs, Biomass yield in substrate (FOS)  %k(5) Ia, Acetate inhibition constant  %k(6) Il, Lactate inhibition constant  %k(7) ms, Cellular maintenance constant  %k(8) Yax, Acetate yield in substrate (FOS)  %k(9) Ylx, Lactate yield in substrate (FOS)  %k(10) ksa, Half-velocity constant with acetate as carbon source  %k(11) ksl, Half-velocity constant with lactate as carbon source  %k(12) betamaxA, Maximum specific production rate of acetate  %k(13) betamaxL, Maximum specific production rate of lactate  %k(14) mumaxA, Maximum growth rate with acetate as carbon source  %k(15) mumaxL, Maximum growth rate with lactate as carbon source  %k(16) YxA, Biomass yield with acetate as carbon source  %k(17) YxL, Biomass yield with lactate as carbon source    %Constitutive equations    %Growth rate with substrate (FOS) as carbon source, also acetate and lactate as inhibitors  mu = k(3)*x(1)/((k(2)+x(1))*(1+(x(2)/k(5)))*(1+(x(3)/k(6))));  %Growth rate with acetate as carbon source, also acetate and lactate as inhibitors  mua = k(14)*x(2)/((k(2)+x(2))*(1+(x(2)/k(5)))*(1+(x(3)/k(6))));  %Growth rate with lactate as carbon source, also acetate and lactate as inhibitors  mul = k(15)*x(3)/((k(2)+x(3))*(1+(x(2)/k(5)))*(1+(x(3)/k(6))));  %Acetate production rate with substrate (FOS) as carbon source  betaA = k(12)*x(1)/(k(10)+x(1));  %Lactate production rate with substrate (FOS) as carbon source  betaL = k(13)*x(1)/(k(11)+x(1));    %Ordinary differential equations    if x(1) > 0    %Substrate (FOS) ODE  sys(1) = -mu*x(4)/k(4) - k(7)*x(4);  %Acetate ODE  sys(2)= betaA*x(4)/k(8);  %Lactate ODE  sys(3)= betaL*x(4)/k(9);  %Living bacteria ODE  sys(4)= mu*x(4)-k(1)*x(4);  %Total bacteria ODE  sys(5)= mu*x(4);  else  %Substrate (FOS) ODE  sys(1)= 0;  %Acetate ODE  sys(2)= -mua*x(4)/k(16);  %Lactate ODE  sys(3)= -mul*x(4)/k(17);  %Living bacteria ODE  sys(4)= mul*x(4)+mua*x(4)-k(1)*x(4);  %Total bacteria ODE  sys(5)= mul*x(4)+mua*x(4);  end |
| --- |

The code used in paired co-cultures analysis is presented in the box below.

| function sys=cocult(t,x,k)  sys = zeros(7,1);    %Variables  %x(1) Substrate (FOS)  %x(2) Acetate  %x(3) Lactate  %x(4) Living bacteria 1  %x(5) Total bacteria 1  %x(4) Living bacteria 2  %x(5) Total bacteria 2    %Parameters    %Bacteria 1 parameters  %k(1) kd, Death rate  %k(2) ks, Half-velocity constant  %k(3) mumax, Maximum growth rate  %k(4) Yxs, Biomass yield in substrate (FOS)  %k(5) Ia, Acetate inhibition constant  %k(6) Il, Lactate inhibition constant  %k(7) ms, Cellular maintenance constant  %k(8) Yax, Acetate yield in substrate (FOS)  %k(9) Ylx, Lactate yield in substrate (FOS)  %k(10) ksa, Half-velocity constant with acetate as carbon source  %k(11) ksl, Half-velocity constant with lactate as carbon source  %k(12) betamaxA, Maximum specific production rate of acetate  %k(13) betamaxL, Maximum specific production rate of lactate  %k(14) mumaxA, Maximum growth rate with acetate as carbon source  %k(15) mumaxL, Maximum growth rate with lactate as carbon source  %k(16) YxA, Biomass yield with acetate as carbon source  %k(17) YxL, Biomass yield with lactate as carbon source    %Bacteria 2 parameters  %k(18) kd, Death rate  %k(19) ks, Half-velocity constant  %k(20) mumax, Maximum growth rate  %k(21) Yxs, Biomass yield in substrate (FOS)  %k(22) Ia, Acetate inhibition constant  %k(23) Il, Lactate inhibition constant  %k(24) ms, Cellular maintenance constant  %k(25) Yax, Acetate yield in substrate (FOS)  %k(26) Ylx, Lactate yield in substrate (FOS)  %k(27) ksa, Half-velocity constant with acetate as carbon source  %k(28) ksl, Half-velocity constant with lactate as carbon source  %k(29) betamaxA, Maximum specific production rate of acetate  %k(30) betamaxL, Maximum specific production rate of lactate  %k(31) mumaxA, Maximum growth rate with acetate as carbon source  %k(32) mumaxL, Maximum growth rate with lactate as carbon source  %k(33) YxA, Biomass yield with acetate as carbon source  %k(34) YxL, Biomass yield with lactate as carbon source    %k(35) ef12 Effect of the bacteria 1 to the bacteria 2  %k(36) ef21 Effect of the bacteria 2 to the bacteria 1    %SSM Restrictions, to avoid NaN’s in every iteration of the parameter optimization  %If the effect of a bacteria is over 100 times, the effect will stay at 100 times maximum  if ((x(6)/k(36)) <= -0.99)  u = -0.99;  else  u = x(6)/k(36);  end    if ((x(4)/k(35)) <= -0.99)  v = -0.99;  else  v = x(4)/k(35);  end    %Constitutive equations  %Bacteria 1    %Growth rate with substrate (FOS) as carbon source, also acetate and lactate as inhibitors  mu1 = k(3)*x(1)/((k(2)+x(1))*(1+(x(2)/k(5)))*(1+(x(3)/k(6)))*(1+u));  %Growth rate with acetate as carbon source, also acetate and lactate as inhibitors  mua1 = k(14)*x(2)/((k(10)+x(2))*(1+(x(2)/k(5)))*(1+(x(3)/k(6)))*(1+u));  %Growth rate with lactate as carbon source, also acetate and lactate as inhibitors  mul1 = k(15)*x(3)/((k(11)+x(3))*(1+(x(2)/k(5)))*(1+(x(3)/k(6))) *(1+u));  %Acetate production rate with substrate (FOS) as carbon source  betaA1 = k(12)*x(1)/(k(2)+x(1));  %Lactate production rate with substrate (FOS) as carbon source  betaL1 = k(13)*x(1)/(k(2)+x(1));    %Bacteria 2  %Growth rate with substrate (FOS) as carbon source, also acetate and lactate as inhibitors  mu2 = k(20)*x(1)/((k(19)+x(1))*(1+(x(2)/k(22)))*(1+(x(3)/k(23)))*(1+v));  %Growth rate with acetate as carbon source, also acetate and lactate as inhibitors  mua2 = k(31)*x(2)/((k(27)+x(2))*(1+(x(2)/k(22)))*(1+(x(3)/k(23)))*(1+v));  %Growth rate with lactate as carbon source, also acetate and lactate as inhibitors  mul2 = k(32)*x(3)/((k(28)+x(3))*(1+(x(2)/k(22)))*(1+(x(3)/k(23)))*(1+v));  %Acetate production rate with substrate (FOS) as carbon source  betaA2 = k(29)*x(1)/(k(19)+x(1));  %Lactate production rate with substrate (FOS) as carbon source  betaL2 = k(30)*x(1)/(k(19)+x(1));  %Ordinary differential equations    if x(1) > 0    %Substrate (FOS) ODE  sys(1) = -mu1*x(4)/k(4) -mu2*x(6)/k(21) - k(7)*x(4)-k(24)*x(6);  %Acetate ODE  sys(2)= betaA1*x(4)/k(8) + betaA2*x(6)/k(25);  %Lactate ODE  sys(3)= betaL1*x(4)/k(9) + betaL2*x(6)/k(26);  %Living bacteria 1 ODE  sys(4)= mu1*x(4)-k(1)*x(4)  %Total bacteria 1 ODE  sys(5)= mu1*x(4);  %Living bacteria 1 ODE  sys(6)= mu2*x(6)-k(18)*x(6);  %Total bacteria 1 ODE  sys(7)= mu2*x(6);  else  %Substrate (FOS) ODE  sys(1)= 0;  %Acetate ODE  sys(2)= -mua1*x(4)/k(16) -mua2*x(6)/k(33);  %Lactate ODE  sys(3)= -mul1*x(4)/k(17) -mul2*x(6)/k(34);  %Living bacteria 1 ODE  sys(4)= mul1*x(4) + mua1*x(4) - k(1)*x(4);  %Total bacteria 1 ODE  sys(5)= mul1*x(4)+mua1*x(4);  %Living bacteria 2 ODE  sys(6)= mul2*x(6) + mua2*x(6) - k(18)*x(6);  %Total bacteria 2 ODE  sys(7)= mul2*x(6)+mua2*x(6);  end |
| --- |

The code used in 4 bacteria co-culture analysis is presented in the box below.

| function sys=consortia(t,x,k)  sys = zeros(11,1);    %Variables  %x(1) Substrate (FOS)  %x(2) Acetate  %x(3) Lactate  %x(4) Living bacteria 1  %x(5) Total bacteria 1  %x(4) Living bacteria 2  %x(5) Total bacteria 2    %Parameters    %Bacteria 1 parameters  %k(1) kd, Death rate  %k(2) ks, Half-velocity constant  %k(3) mumax, Maximum growth rate  %k(4) Yxs, Biomass yield in substrate (FOS)  %k(5) Ia, Acetate inhibition constant  %k(6) Il, Lactate inhibition constant  %k(7) ms, Cellular maintenance constant  %k(8) Yax, Acetate yield in substrate (FOS)  %k(9) Ylx, Lactate yield in substrate (FOS)  %k(10) ksa, Half-velocity constant with acetate as carbon source  %k(11) ksl, Half-velocity constant with lactate as carbon source  %k(12) betamaxA, Maximum specific production rate of acetate  %k(13) betamaxL, Maximum specific production rate of lactate  %k(14) mumaxA, Maximum growth rate with acetate as carbon source  %k(15) mumaxL, Maximum growth rate with lactate as carbon source  %k(16) YxA, Biomass yield with acetate as carbon source  %k(17) YxL, Biomass yield with lactate as carbon source    %Bacteria 2 parameters  %k(18) kd, Death rate  %k(19) ks, Half-velocity constant  %k(20) mumax, Maximum growth rate  %k(21) Yxs, Biomass yield in substrate (FOS)  %k(22) Ia, Acetate inhibition constant  %k(23) Il, Lactate inhibition constant  %k(24) ms, Cellular maintenance constant  %k(25) Yax, Acetate yield in substrate (FOS)  %k(26) Ylx, Lactate yield in substrate (FOS)  %k(27) ksa, Half-velocity constant with acetate as carbon source  %k(28) ksl, Half-velocity constant with lactate as carbon source  %k(29) betamaxA, Maximum specific production rate of acetate  %k(30) betamaxL, Maximum specific production rate of lactate  %k(31) mumaxA, Maximum growth rate with acetate as carbon source  %k(32) mumaxL, Maximum growth rate with lactate as carbon source  %k(33) YxA, Biomass yield with acetate as carbon source  %k(34) YxL, Biomass yield with lactate as carbon source    %Bacteria 3 parameters  %k(35) kd, Death rate  %k(36) ks, Half-velocity constant  %k(37) mumax, Maximum growth rate  %k(38) Yxs, Biomass yield in substrate (FOS)  %k(39) Ia, Acetate inhibition constant  %k(40) Il, Lactate inhibition constant  %k(41) ms, Cellular maintenance constant  %k(42) Yax, Acetate yield in substrate (FOS)  %k(43) Ylx, Lactate yield in substrate (FOS)  %k(44) ksa, Half-velocity constant with acetate as carbon source  %k(45) ksl, Half-velocity constant with lactate as carbon source  %k(46) betamaxA, Maximum specific production rate of acetate  %k(47) betamaxL, Maximum specific production rate of lactate  %k(48) mumaxA, Maximum growth rate with acetate as carbon source  %k(49) mumaxL, Maximum growth rate with lactate as carbon source  %k(50) YxA, Biomass yield with acetate as carbon source  %k(51) YxL, Biomass yield with lactate as carbon source    %Bacteria 2 parameters  %k(52) kd, Death rate  %k(53) ks, Half-velocity constant  %k(54) mumax, Maximum growth rate  %k(55) Yxs, Biomass yield in substrate (FOS)  %k(56) Ia, Acetate inhibition constant  %k(57) Il, Lactate inhibition constant  %k(58) ms, Cellular maintenance constant  %k(59) Yax, Acetate yield in substrate (FOS)  %k(60) Ylx, Lactate yield in substrate (FOS)  %k(61) ksa, Half-velocity constant with acetate as carbon source  %k(62) ksl, Half-velocity constant with lactate as carbon source  %k(63) betamaxA, Maximum specific production rate of acetate  %k(64) betamaxL, Maximum specific production rate of lactate  %k(65) mumaxA, Maximum growth rate with acetate as carbon source  %k(66) mumaxL, Maximum growth rate with lactate as carbon source  %k(67) YxA, Biomass yield with acetate as carbon source  %k(68) YxL, Biomass yield with lactate as carbon source  %k(69) ef12 Effect of the bacteria 1 to the bacteria 2  %k(70) ef13 Effect of the bacteria 1 to the bacteria 3  %k(71) ef14 Effect of the bacteria 1 to the bacteria 4  %k(72) ef21 Effect of the bacteria 2 to the bacteria 1  %k(73) ef23 Effect of the bacteria 2 to the bacteria 3  %k(74) ef24 Effect of the bacteria 2 to the bacteria 4  %k(75) ef31 Effect of the bacteria 3 to the bacteria 1  %k(76) ef32 Effect of the bacteria 3 to the bacteria 2  %k(77) ef34 Effect of the bacteria 3 to the bacteria 4  %k(78) ef41 Effect of the bacteria 4 to the bacteria 1  %k(79) ef42 Effect of the bacteria 4 to the bacteria 2  %k(80) ef43 Effect of the bacteria 4 to the bacteria 3    %Constitutive equations  %Bacteria 1    %Growth rate with substrate (FOS) as carbon source, also acetate and lactate as inhibitors  mu1 = k(3)*x(1)/((k(2)+x(1))*(1+(x(2)/k(5)))*(1+(x(3)/k(6)))*(1+(x(6)/k(72)))*(1+(x(8)/k(75)))*(1+(x(10)/k(78))));  %Growth rate with acetate as carbon source, also acetate and lactate as inhibitors  mua1 = k(14)*x(2)/((k(2)+x(2))*(1+(x(2)/k(5)))*(1+(x(3)/k(6)))*(1+(x(6)/k(72)))*(1+(x(8)/k(75)))*(1+(x(10)/k(78))));  %Growth rate with lactate as carbon source, also acetate and lactate as inhibitors  mul1 = k(15)*x(3)/((k(2)+x(3))*(1+(x(2)/k(5)))*(1+(x(3)/k(6)))*(1+(x(6)/k(72)))*(1+(x(8)/k(75)))*(1+(x(10)/k(78))));  %Acetate production rate with substrate (FOS) as carbon source  betaA1 = k(12)*x(1)/(k(2)+x(1));  %Lactate production rate with substrate (FOS) as carbon source  betaL1 = k(13)*x(1)/(k(2)+x(1));    %Bacteria 2  %Growth rate with substrate (FOS) as carbon source, also acetate and lactate as inhibitors  mu2 = k(20)*x(1)/((k(19)+x(1))*(1+(x(2)/k(22)))*(1+(x(3)/k(23)))*(1+(x(4)/k(69)))*(1+(x(8)/k(76)))*(1+(x(10)/k(79))));  %Growth rate with acetate as carbon source, also acetate and lactate as inhibitors  mua2 = k(31)*x(2)/((k(19)+x(2))*(1+(x(2)/k(22)))*(1+(x(3)/k(23)))*(1+(x(4)/k(69)))*(1+(x(8)/k(76)))*(1+(x(10)/k(79))));  %Growth rate with lactate as carbon source, also acetate and lactate as inhibitors  mul2 = k(32)*x(3)/((k(19)+x(3))*(1+(x(2)/k(22)))*(1+(x(3)/k(23)))*(1+(x(4)/k(69)))*(1+(x(8)/k(76)))*(1+(x(10)/k(79))));  %Acetate production rate with substrate (FOS) as carbon source  betaA2 = k(29)*x(1)/(k(19)+x(1));  %Lactate production rate with substrate (FOS) as carbon source  betaL2 = k(30)*x(1)/(k(19)+x(1));  %Bacteria 3  %Growth rate with substrate (FOS) as carbon source, also acetate and lactate as inhibitors  mu3 = k(37)*x(1)/((k(36)+x(1))*(1+(x(2)/k(39)))*(1+(x(3)/k(40)))*(1+(x(4)/k(70)))*(1+(x(6)/k(73)))*(1+(x(10)/k(80))));  %Growth rate with acetate as carbon source, also acetate and lactate as inhibitors  mua3 = k(48)*x(2)/((k(36)+x(2))*(1+(x(2)/k(39)))*(1+(x(3)/k(40)))*(1+(x(4)/k(70)))*(1+(x(6)/k(73)))*(1+(x(10)/k(80))));  %Growth rate with lactate as carbon source, also acetate and lactate as inhibitors  mul3 = k(49)*x(3)/((k(36)+x(3))*(1+(x(2)/k(39)))*(1+(x(3)/k(40)))*(1+(x(4)/k(70)))*(1+(x(6)/k(73)))*(1+(x(10)/k(80))));  %Acetate production rate with substrate (FOS) as carbon source  betaA3 = k(46)*x(1)/(k(44)+x(1));  %Lactate production rate with substrate (FOS) as carbon source  betaL3 = k(47)*x(1)/(k(45)+x(1));  %Bacteria 4  %Growth rate with substrate (FOS) as carbon source, also acetate and lactate as inhibitors  mu4 = k(54)*x(1)/((k(53)+x(1))*(1+(x(2)/k(56)))*(1+(x(3)/k(57)))*(1+(x(4)/k(71)))*(1+(x(6)/k(74)))*(1+(x(8)/k(77))));  %Growth rate with acetate as carbon source, also acetate and lactate as inhibitors  mua4 = k(65)*x(2)/((k(53)+x(2))*(1+(x(2)/k(56)))*(1+(x(3)/k(57)))*(1+(x(4)/k(71)))*(1+(x(6)/k(74)))*(1+(x(8)/k(77))));  %Growth rate with lactate as carbon source, also acetate and lactate as inhibitors  mul4 = k(66)*x(3)/((k(53)+x(3))*(1+(x(2)/k(56)))*(1+(x(3)/k(57)))*(1+(x(4)/k(71)))*(1+(x(6)/k(74)))*(1+(x(8)/k(77))));  %Acetate production rate with substrate (FOS) as carbon source  betaA4 = k(63)*x(1)/(k(61)+x(1));  %Lactate production rate with substrate (FOS) as carbon source  betaL4 = k(64)*x(1)/(k(62)+x(1));  %Ordinary differential equations    if x(1) > 0    %Substrate (FOS) ODE  sys(1) = -mu1*x(4)/k(4) -mu2*x(6)/k(21) -mu3*x(8)/k(38) -mu4*x(10)/k(55)- k(7)*x(4)-k(24)*x(6)- k(41)*x(8)-k(58)*x(10);  %Acetate ODE  sys(2) = betaA1*x(4)/k(8) + betaA2*x(6)/k(25) + betaA3*x(8)/k(42) + betaA4*x(10)/k(59);  %Lactate ODE  sys(3) = betaL1*x(4)/k(9) + betaL2*x(6)/k(26) + betaL3*x(8)/k(43) + betaL4*x(10)/k(60);  %Living bacteria 1 ODE  sys(4) = mu1*x(4)-k(1)*x(4);  %Total bacteria 1 ODE  sys(5) = mu1*x(4);    %Living bacteria 2 ODE  sys(6) = mu2*x(6)-k(18)*x(6);  %Total bacteria 2 ODE  sys(7) = mu2*x(6);  %Living bacteria 3 ODE  sys(8) = mu3*x(8)-k(35)*x(8);    %Total bacteria 3 ODE  sys(9) = mu3*x(8);  %Living bacteria 4 ODE  sys(10)= mu4*x(10)-k(52)*x(10);  %Total bacteria 4 ODE  sys(11)= mu4*x(10);  else  %Substrate (FOS) ODE  sys(1)= 0;  %Acetate ODE  sys(2)= -mua1*x(4)/k(16) -mua2*x(6)/k(33)-mua3*x(8)/k(50) -mua4*x(10)/k(67);  %Lactate ODE  sys(3)= -mul1*x(4)/k(17) -mul2*x(6)/k(34)-mul3*x(8)/k(51) -mul4*x(10)/k(68);  %Living bacteria 1 ODE  sys(4)= mul1*x(4) + mua1*x(4) - k(1)*x(4);  %Total bacteria 1 ODE  sys(5)= mul1*x(4)+mua1*x(4);  %Living bacteria 2 ODE  sys(6)= mul2*x(6) + mua2*x(6) - k(18)*x(6);  %Total bacteria 2 ODE  sys(7)= mul2*x(6)+mua2*x(6);  %Living bacteria 3 ODE  sys(8)= mul3*x(8) + mua3*x(8) - k(35)*x(8);  %Total bacteria 3 ODE  sys(9)= mul3*x(8)+mua3*x(8);  %Living bacteria 4 ODE  sys(10)= mul4*x(10) + mua4*x(10) - k(52)*x(10);  %Total bacteria 4 ODE  sys(11)= mul4*x(10)+mua4*x(10);  end |
| --- |

**References**

Andrews, J. F. (1968). A mathematical model for the continuous culture of microorganisms utilizing inhibitory substrates. Biotechnol. Bioeng., 10: 707–723. doi:10.1002/bit.260100602

Egea, J.A., Martí, R., & Banga, J.R. (2010). An evolutionary method for complex-process optimization. Computers and Operations Research 37(2):315-324.

Rodriguez-Fernandez, M., J. A. Egea and J. R. Banga (2006) Novel Metaheuristic for Parameter Estimation in Nonlinear Dynamic Biological Systems. BMC Bioinformatics 7:483

Sacher, J., Saa, P., Cárcamo, M., López, J., Gelmi, C. A., and Pérez-Correa, R. (2011). Improved calibration of a solid substrate fermentation model. *Electron. J. Biotechnol.* 14. doi:10.2225/vol14-issue5-fulltext-7.

# Supplementary Tables

## Table S1: Error calculated (equation 10, supplementary material) in each parameter fit between the model and the experimental data in mono-culture (A); paired co-cultures (B) and four—bacteria microplates and bioreactor (C).

| A) |  | B) |  | C) |  |
| --- | --- | --- | --- | --- | --- |
| Mono-culture | Error function  value | Co-culture | Error function  value | Four-bacteria  co-culture | Error function  value |
| Bi | 8.90E+05 | Bi-Bv | 1.42E+05 | Bi-Bv-Ec-La Plates | 5.49E+03 |
| Bv | 3.13E+02 | Bi-Ec | 4.51E+03 | Bi-Bv-Ec-La Bioreactor | 8.58E+01 |
| Ec | 2.73E+02 | Bi-La | 8.34E+02 |  |  |
| La | 3.37E+04 | Bv-Ec | 1.96E+05 |  |  |
|  |  | Bv-La | 1.80E+02 |  |  |
|  |  | La-Ec | 3.51E+02 |  |  |
